# Optimization of Outflow-Tract Ventricular Arrhythmia Ablation Using a Universal Right Ventricle Model

**DOI:** 10.3390/jcdd12090323

**Published:** 2025-08-24

**Authors:** Krystian Szkoła, Łukasz Zarębski, Paweł Turek, Marian Futyma, Łukasz Wiśniowski, Piotr Futyma

**Affiliations:** 1 Clinical Electrophysiology, St. Joseph’s Heart Rhythm Center, 35-623 Rzeszów, Poland; k.szkola93@gmail.com (K.S.); lukasz.zarebski@interia.pl (Ł.Z.); marian.futyma@wp.pl (M.F.); 2 Department of Cardiology and Interventional Cardiology, St. Padre Pio’s Hospital in Przemyśl, 37-700 Przemyśl, Poland; dr.lukasz.wisniowski@gmail.com; 3 Faculty of Medicine, University of Rzeszów, 35-310 Rzeszów, Poland; 4 Faculty of Mechanical Engineering and Aeronautics, Rzeszów University of Technology, 35-959 Rzeszów, Poland; pturek@prz.edu.pl

**Keywords:** ventricular arrhythmia, universal modeling, 3D mapping, right ventricle outflow tract, additive manufacturing, CAD modeling, reverse engineering

## Abstract

**Introduction:** The radiofrequency catheter ablation (RFCA) of ventricular arrhythmias (VAs) originating from the right ventricular outflow tract (RVOT) is a well-established therapy. Traditionally, RFCA is guided using electroanatomical 3D mapping systems involving manual catheter navigation within cardiac chambers. While effective, this approach may be time-consuming, and it carries a potential risk of cardiac wall perforation. Although the risk is low, it cannot be underestimated. Therefore, alternative mapping methods are sought to reduce procedural times and improve the overall efficiency of RVOT-VAs ablation. **Aim:** To evaluate the safety, feasibility, and efficacy of a universal RVOT 3D model implementation for the ablation of idiopathic RVOT-VAs. **Methods:** Consecutive patients undergoing VA ablation supported with a universal RVOT 3D model (3D-MODEL group) were included in the study. The RVOT universal model in this group was created by processing DICOM images for the improved segmentation of anatomical structures, followed by production using 3D printing technology. Patients who underwent classic endocardial electroanatomical mapping served as controls (EAM group). **Results:** A total of 228 patients were included in the study (143 women, age 50 ± 17 years): 149 in the 3D-MODEL group and 79 in the EAM group. The acute complete elimination of clinical VAs was achieved for 133 (89%) of patients in the 3D-MODEL group vs. 65 (82%) in the EAM group (*p* = 0.14). The procedural time was significantly shorter in the 3D-MODEL group compared to the EAM group (38 ± 14 min vs. 80 ± 39 min, *p* < 0.001). A significant difference was also observed in the radiofrequency time between the 3D-MODEL and EAM groups (251 ± 176 s vs. 503 ± 425 s, *p* < 0.001). No significant difference in fluoroscopy time was found between the groups (284 ± 167 s vs. 260 ± 327 s, *p* = 0.49). Two cases of cardiac tamponade occurred, both in patients from the EAM group. During follow-up, lasting 14 ± 10 months, 87% of patients in the 3D-MODEL group and 75% in the EAM group remained arrhythmia-free (*p* = 0.45). **Conclusions:** The use of universal RVOT 3D modeling is a feasible, safe, and effective alternative to classic electroanatomical mapping in the ablation of idiopathic RVOT-VAs.

## 1. Introduction

The right ventricular outflow tract (RVOT) is one of the most common origins of ventricular arrhythmias (VAs) [1]. While often benign, VAs can be symptomatic and may lead to heart failure or, in very rare cases, sudden cardiac death [2,3]. Radiofrequency catheter ablation (RFCA) is a well-established therapy for RVOT-VAs [4]. Traditional electroanatomical mapping (EAM) systems, based on either impedance or electromagnetic fields, enable the creation of a three-dimensional reconstruction of the RVOT. However, EAM requires specialized and expensive equipment and can be time-consuming [5]. Furthermore, endocardial mapping with an ablation catheter carries a potential risk of mechanical perforation [6,7]. In this study, we describe and evaluate a novel approach involving a universal three-dimensional model of the RVOT [8]. The model is based on an advanced version of the 4D extended cardiac-torso (XCAT, Duke University) phantom, which was originally developed to support the evaluation and optimization of cardiac imaging techniques, particularly computed tomography. In addition to its imaging applications, it also enables procedural planning and simulation of catheter-based interventions involving the VA ablation. Our aim was to assess the safety and efficacy of RFCA, guided by the universal 3D RVOT model, based on XCAT, and merged with fluoroscopy, in comparison to classic EAM.

## 2. Materials and Methods

Consecutive patients undergoing RVOT ablation for symptomatic premature ventricular contractions (PVCs) and ventricular tachycardia (VT) from two centers between May 2012 and April 2024 were included in the study. The inclusion criteria were as follows: age ≥ 18 years, documented ventricular arrhythmia originating from the RVOT, and no prior ablations within the RVOT. Only patients in whom ablation was performed exclusively within the RVOT were included. Cases in which ablation was performed both in the RVOT and in the left ventricular outflow tract (LVOT) during the same procedure were excluded. Baseline clinical characteristics, including age, sex, clinical presentation, and findings from a standard 12-lead electrocardiogram (ECG) or a 24 h, 12-lead Holter ECG, were recorded. All procedures were conducted under conscious sedation and local anesthesia. Whenever possible, antiarrhythmic medications were discontinued for two to five half-lives before the procedure, in accordance with each center’s standard protocol for VA ablation.

### 2.1. 3D Reconstruction of the RVOT Model

**Figure 1:**
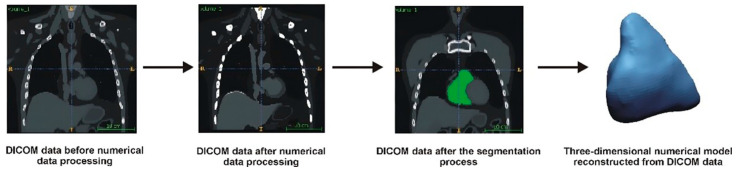
Diagram of the procedure for reconstructing the geometry of the anatomical model.

The first step in developing the numerical RVOT model was a data-filtering process to remove noise from the Digital Imaging and Communications in Medicine (DICOM) images. Next, the spatial resolution of the DICOM images was enhanced using the Lanczos interpolation method [9,10]. This method involved determining additional pixels based on the intensity of neighboring pixels. By applying this interpolation process, we could accelerate the extraction of the anatomical structures while improving the accuracy of the geometry restoration, particularly by minimizing step artifacts in the DICOM images. After the interpolation, additional filtering was conducted to sharpen the boundaries between the anatomical structures and surrounding tissues. Following this, a segmentation process was performed, which involved extracting the anatomical structures from the entire volumetric data set using the global thresholding method [10]. A lower segmentation threshold of 50 on the Hounsfield scale was applied to delineate the contours of the segmented structure. To visualize the 3D model, a voxel-based method utilizing the Marching Cubes algorithm was used. The final output was a digital 3D model saved in a Stereolithography (STL) file [11]. Figure 1 illustrates a general schematic of the geometry reconstruction process.

### 2.2. Additive Manufacturing of the RVOT Model

**Figure 2:**
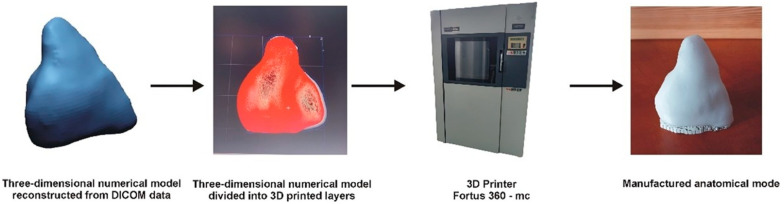
Schematic diagram of the 3D printing process of an anatomical model.

The model was manufactured using the FDM additive method, part of material extrusion technology [12,13]. Fused deposition modeling (FDM), also referred to as fused filament fabrication (FFF), is an additive manufacturing technique based on material extrusion. In this process, a thermoplastic filament is heated and precisely extruded through a nozzle, depositing material layer by layer along a programmed path to construct the final three-dimensional structure. A Fortus 360-mc printer from Stratasys was utilized for 3D printing the anatomical model. This printer features movable print heads and a worktable. The thermoplastic material is supplied to the print heads from cassettes, where it is melted and pumped into heated print tips. The numerically controlled machine alternately deposits the model and support material onto the worktable according to the generated layers, building the complete model layer by layer. Before starting the FDM printing process, the digital model is loaded into the Insight 7.0 software. The software allowed the model to be divided into layers and generated supports. A complete fill was applied to the entire model when subdividing the model into layers. For the parameter that defines the style of visible surfaces, the enhanced option was selected to achieve the best possible representation of the manufactured surface. The 3D-printed model was created using PC-10 material and T12 print heads (Stratasys, Ltd., Eden Prairie, MN, USA), maintaining a thickness of 0.178 mm. A general schematic illustrating the manufacturing process of the model using the FDM additive method is presented in Figure 2.

### 2.3. Universal Model of RVOT During EP Procedure

**Figure 3:**
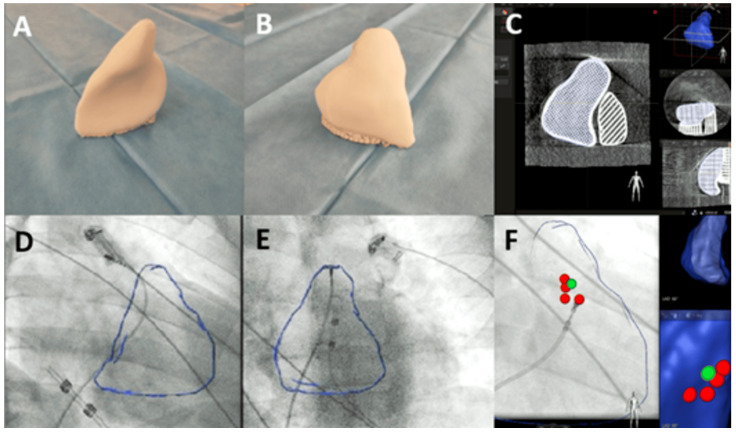
(**A**,**B**): 3D printed model of the right ventricular outflow tract (RVOT) based on XCAT. (**C**): DICOM image of the RVOT 3D model. (**D**,**E**): merging of the RVOT 3D model with fluoroscopy imaging in RAO 30° (**D**) and LAO 30° (**E**) projections. (**F**): ablation tags localized at the septal sites of the RVOT (red spots refer to sites of RF applications, green spot refers to the site of the earliest activation).

In the study group (3D-MODEL group), mapping was guided by a universal three-dimensional RVOT model based (derived from XCAT DICOM) merged with fluoroscopic projections (RAO 30° and LAO 30°) via the EP Navigator™ mapping system (Philips, Best, the Netherlands) (Figure 3). All patients were mapped using the same RVOT model. Arrhythmia localization was based on a combination of 12-lead ECG morphology analysis, pacemapping, and limited intracardiac activation mapping within predefined anatomical zones of interest guided by the universal RVOT model. No anatomical point-by-point EAM reconstruction was performed. In the control group (EAM group), patients underwent classic endocardial mapping with an ablation catheter connected to a three-dimensional EAM system: ENSITE™ (Abbott, St Paul, MN, USA) or CARTO™ (Biosense Webster, Diamond Bar, CA, USA). In cases with infrequent PVCs during the procedure, arrhythmia induction was attempted using intravenous boluses of salbutamol (0.125 mg) or ventricular/atrial burst pacing. General schematic illustrations showing the use of the 3D-printed model of the RVOT during the EP procedure are presented in Figure 3.

### 2.4. Catheter Ablation

All ablations were performed using standard RF generators—either the Ampere™ system (St. Jude Medical, St. Paul, MN, USA) or the EP Shuttle (Stockert, Freiburg, Germany). The following catheters were used: Flexibility™ (Abbott, St. Paul, MN, USA), Blazer™ (Boston Scientific, Marlborough, MA, USA), (APT, Shenzen, China), Navistar Thermocool™ (Biosense-Webster, Diamond Bar, CA, USA), AlCath FullCricle (Biotronik, Berlin, Germany), Triguy 4mm™, and Triguy CoolTip™ (APT, Shenzen, China). Ablation was performed using non-irrigated 4 mm tip catheters (in the majority of 3D-MODEL group cases) or open-irrigated catheters (predominantly in the EAM group). Non-irrigated catheters operated in temperature-controlled mode with a target electrode temperature of approximately 60–65 °C, while irrigated catheters were used in power-controlled mode. Ablation parameters, including power and temperature, were selected at the operator’s discretion and the catheter manufacturer’s recommendations. Contact-force sensing catheters were not used in this study.

The acute procedural endpoint was defined as the absence of clinical VAs during a 30 min observation period following the final RF application, including after the administration of intravenous salbutamol boluses. The secondary endpoint was freedom from ventricular arrhythmia during the follow-up.

### 2.5. Follow-Up

The long-term efficacy of PVC/VT ablation was evaluated based on regular outpatient follow-up visits. In the vast majority of patients, rhythm monitoring included a 12-lead ECG and a 24 h Holter. In the remaining cases, follow-up relied on alternative methods, such as inpatient telemetry during hospitalization, outpatient rhythm monitoring, device interrogation (if applicable), and clinical evaluation during follow-up visits. The first post-ablation visit was scheduled between one and three months after discharge, depending on each center’s routine practice and the patient’s clinical status. Subsequent follow-up visits were performed at intervals determined by the treating physician or as clinically indicated. Arrhythmia elimination was defined as a reduction of ≥80% in the PVC burden, whereas arrhythmia suppression was defined as a reduction in the PVC burden between 50% and 80% during the follow-up period.

### 2.6. Statistical Analysis

For statistical analysis, continuous variables were expressed as means ± standard deviations, and categorical variables were reported as counts and percentages. Categorical variables were compared using chi-square or Fisher’s exact tests, with Yates correction applied when appropriate. Comparisons between continuous variables were made using two-tailed Student’s t-tests. Kaplan–Meier survival analysis was conducted to evaluate the recurrence rates of PVCs and VT and the time to recurrence. A *p*-value < 0.05 was considered statistically significant.

## 3. Results

A total of 228 patients (143 women; age: 50 ± 16 years) were included in the study, with 149 assigned to the 3D-MODEL group and 79 to the EAM group. The procedural time was significantly shorter in the 3D-MODEL group compared to the EAM group (39 ± 15 min vs. 81 ± 39 min, *p* < 0.001). Fluoroscopy times did not differ significantly between the groups (276 ± 324 s vs. 297 ± 181 s, *p* = 0.52). The mean power applied during RF ablation was significantly higher in the 3D-MODEL group than in the EAM group (41 ± 9 W vs. 36 ± 8 W, *p* < 0.001). In the 3D-MODEL group, 123 patients (83%) had ventricular arrhythmia originating from the septal portion of the RVOT, compared to 48 patients (63%) in the EAM group (*p* = 0.001). In the remaining 26 (17%) patients of the 3D-MODEL group and 31 patients (39%) in the EAM group, the arrhythmia originated from the RVOT free wall.

The acute elimination of clinical PVCs and the non-inducibility of VT were achieved in 132 patients (89%) in the 3D-MODEL group and in 65 patients (82%) in the EAM group. Arrhythmia suppression, defined as a reduction in PVC frequency to less than one ectopic beat per minute, was achieved in an additional 17 patients (11%) and 11 patients (14%), respectively. In the EAM group, the remaining three patients (4%) showed no acute effect. The difference in acute procedural success between the groups was not statistically significant (*p* = 0.14). There was no significant difference in acute ablation success between the 3D-MODEL group and the EAM group within the PVC subcohort (89% vs. 81%, *p* = 0.10) and the VT subcohort (91% vs. 92%, *p* = 0.95). Table 1 shows clinical characteristics in the 3D-MODEL and EAM groups. A comparison of procedural characteristics between the 3D-MODEL and EAM groups is presented in Table 2.

Major complications occurred in two patients from the EAM group, both of whom experienced cardiac tamponade requiring pericardiocentesis. There was one episode of mechanically induced complete atrioventricular block during EAM mapping, which required temporary pacing and resolved spontaneously after a short period of observation. No major complications were observed in the 3D-MODEL group.

**Figure 4:**
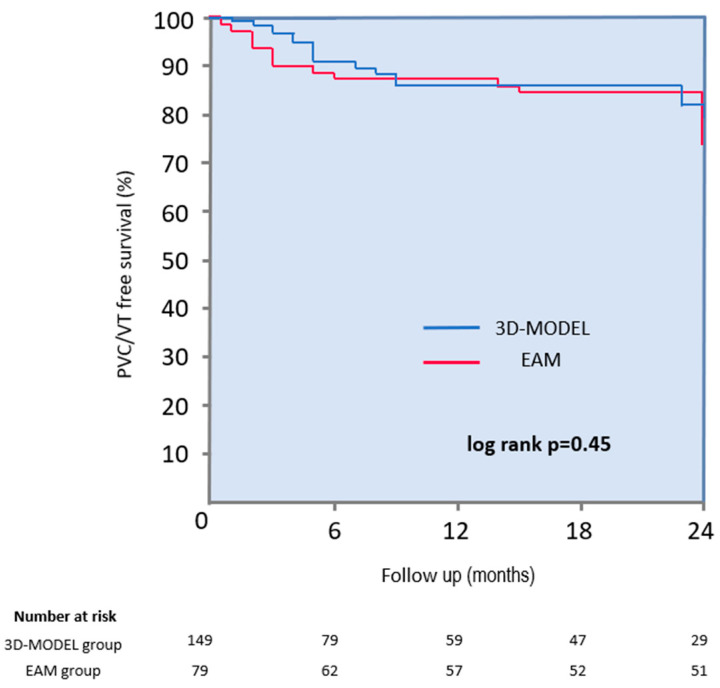
Kaplan–Meier survival curves demonstrating freedom from the clinical PVCs and VT in 3D-MODEL and EAM groups after RF ablation. PVCs = premature ventricular complexes; EAM = electroanatomical mapping; VT = ventricular tachycardia. “Number at risk” under the Kaplan–Meier curves refers to the number of study participants who are still under observation and have not yet experienced the endpoint event (e.g., death or the recurrence of clinical arrhythmia) at a given time point.

During a mean follow-up period of 14 ± 10 months, 82% of patients remained free from arrhythmia (87% in the 3D-MODEL group vs. 75% in the EAM group; log-rank *p* = 0.45) (Figure 4). A subgroup analysis revealed no significant differences in long-term efficacy between groups: in patients with PVCs, arrhythmia-free survival was 83% in the 3D-MODEL group vs. 70% in the EAM group (*p* = 0.90), and in patients with VT, it was 91% vs. 67%, respectively (*p* = 0.37). Three patients were lost to follow-up. A twenty-four-hour Holter ECG was performed for over 90% of patients during follow-up. Although Holter monitoring is considered the most important for assessing the post-ablation arrhythmia burden, it was not obtained from all patients due to the retrospective nature of the study. In selected patients, long-term procedural efficacy was assessed based on consistent clinical indicators—such as stable sinus rhythm documented in repeated 12-lead ECGs and complete resolution of symptoms—particularly when the arrhythmia had been previously frequent and clearly evident on every baseline ECG.

## 4. Discussion

### 4.1. Electroanatomical Mapping and Its Limitations

Classic techniques for the three-dimensional reconstruction of cardiac chambers and the localization of arrhythmogenic foci typically rely on EAM. This approach combines real-time anatomical reconstruction with electrical activation mapping, enabling the identification of ectopic foci. However, several EAM-related factors may impact procedural outcomes.

First, the accuracy of EAM is contingent upon the density of mapping points and the expertise of the operators and support staff, introducing inter-procedural variability [5,14]. Achieving high-density maps often necessitates extensive catheter manipulation, which can prolong the procedure and increase the risk of complications such as mechanical perforation. Moreover, spatial accuracy may be compromised by catheter instability and respiratory motion, both of which can distort the reconstructed map.

Catheter-induced ectopy is another potential drawback of EAM, as it may result in the recording of non-clinical premature beats, leading to the inaccurate localization of the arrhythmogenic substrate and suboptimal ablation outcomes [15]. Additionally, intraprocedural map shifts—caused by patient movement, physiological changes, or system limitations—may require remapping and prolong the procedure. Moreover, if such an error remains undetected, these shifts can result in mapping error and lead to ineffective ablation. The mechanical suppression of PVCs during catheter manipulation should also be considered a double-edged sword. While it may complicate localization of the clinical arrhythmia—particularly when significant catheter-induced trauma alters the arrhythmic substrate or transiently eliminates the ectopy—it can also serve as an indirect marker of proximity to the arrhythmogenic focus, especially when suppression occurs reproducibly at a specific site [16,17,18].

### 4.2. Anatomy of the RVOT

The RVOT is a common origin site of idiopathic VAs due to its distinct anatomical and electrophysiological characteristics. It is a thin-walled, tubular structure situated between the supraventricular crest and the pulmonary valve, representing a transition zone from myocardial to fibrous tissue [1]. Myocardial fibers within the RVOT are arranged circumferentially in the subepicardium and longitudinally in the subendocardium, contributing to its unique conduction properties and susceptibility to arrhythmogenesis [19]. Furthermore, the RVOT free wall is subject to greater shear stress than the septal region, which may promote structural remodeling and enhance arrhythmogenic potential in this area. Despite individual anatomical variability, the RVOT exhibits relatively consistent landmarks, making it a well-defined and frequently targeted region for catheter ablation, with high procedural success rates reported in the literature [20].

### 4.3. Universal Three-Dimensional Model of the RVOT

In our study, a simplified mapping strategy was used. Three-dimensional chamber reconstruction was based on a universal RVOT model, while localization of the arrhythmogenic focus relied primarily on a combination of detailed surface ECG analysis and intracardiac mapping techniques limited to the area of interest. Initially, a 12-lead ECG was used to determine the approximate site of arrhythmia origin within the RVOT, differentiating between septal and free wall locations based on QRS morphology [21]. This was preceded by brief activation mapping of the targeted region and followed by pacemapping to confirm arrhythmia origin.

Although the universal model does not incorporate individual anatomical variability, in idiopathic RVOT arrhythmias, the origin is typically confined to reproducible anatomical zones. The predefined structure thus enables efficient, targeted mapping limited to clinically relevant regions, potentially reducing catheter manipulation time and mechanical irritation. While classic EAM systems are associated with high per-procedure costs related to equipment maintenance, licensing, and disposables, the universal RVOT model—once generated—can be reused and shared across centers, limiting the need for repetitive imaging and modeling [22,23]. Although no formal cost analysis was conducted, the universal RVOT model—once developed—may offer substantial per-procedure cost savings.

In a minority of cases, arrhythmogenic foci may be intramural and require combined mapping of both the RVOT and LVOT for effective localization and ablation. However, these cases are relatively uncommon among idiopathic RVOT ventricular arrhythmias. Intramural origins can often be anticipated pre-procedurally through specific ECG characteristics [24] or by identifying areas of broad early activation within the RVOT [25]. Such cases may require advanced mapping and ablation strategies such as microcatheter or wire-based mapping or sequential unipolar, alcohol, or bipolar ablation [26,27]. While these approaches are relevant for a small subset of patients, they fall beyond the scope of the present study. Our findings support the use of a universal 3D RVOT model as an effective first-line strategy, with additional mapping of the LVOT reserved for carefully selected cases in which an intramural origin is suspected.

Moreover, in the absence of clear electrocardiographic or procedural indicators of a left-sided origin, the routine extension of the procedure to the LVOT may not be justified. Several studies have demonstrated that idiopathic ventricular arrhythmias may exhibit delayed suppression following ablation, with spontaneous resolution occurring even as late as days or months after ablation [28,29]. Considering this, a more conservative procedural strategy may be preferable—one that includes post-procedural observation and reserves LVOT mapping for a potential second-stage procedure if arrhythmia recurrence is documented. Such an approach may avoid unnecessary left-sided access and reduce procedural risk in cases where RVOT ablation alone ultimately proves effective.

### 4.4. Advantages of Universal RVOT Mapping Model

To our knowledge, this is the first study to evaluate the feasibility of using a universal RVOT mapping model to guide catheter ablation. The predefined anatomical guidance enabled more rapid identification of arrhythmogenic foci, limiting the need for extensive point-by-point electroanatomical mapping. This results in a significant reduction in procedural time compared to conventional EAM while maintaining comparable rates of acute procedural success. Moreover, by minimizing catheter manipulation, the use of a universal 3D anatomical model may also reduce the risk of mechanical irritation or cardiac perforation. Although traditional 3D mapping systems are intended to reduce fluoroscopy exposure, our study did not show a significant difference in fluoroscopy time between the universal model and EAM groups [30,31,32], likely due to the continuous use of fluoroscopy for model alignment and catheter navigation in the 3D-MODEL group.

Additionally, the universal model enabled the use of standard non-irrigated ablation catheters, as procedures were performed without the need for 3D electroanatomical reconstruction. In contrast, open-irrigated catheters were required in the EAM group due to compatibility with mapping systems such as Ensite™ or Carto™, which rely on location sensors. To the best of our knowledge, non-irrigated catheters equipped with such sensors are not available in the Polish healthcare system. Importantly, the ablation of idiopathic RVOT arrhythmias typically does not require open-irrigated catheters, and the use of a universal 3D model facilitates a simplified and cost-effective workflow based on fluoroscopic guidance.

This study is a pilot evaluation of a universal anatomical modeling technique. In the future, more individualized approaches—potentially incorporating non-invasive imaging data such as RVOT diameters obtained via transthoracic echocardiography—may enable the development of patient-specific models to further optimize the catheter ablation of idiopathic RVOT arrhythmias.

### 4.5. Limitations

First, this was a retrospective study. To validate our findings, prospective studies involving a larger number of centers should be conducted. Additionally, the observed differences in clinical outcomes may, at least in part, be attributable to variations in the positioning of the dispersive patch, which can affect lesion formation and procedural efficacy [33]. Lastly, anatomical variability in RVOT dimensions and trabecular structures may impact mapping accuracy.

## 5. Conclusions

The use of a universal 3D-MODEL of RVOT is a feasible, safe, and effective alternative to classic catheter mapping for the ablation of idiopathic RVOT-VAs. Future research should focus on validating these results in larger, prospective, controlled trial groups and on comparing universal modeling with different types of EAM techniques.

## Figures and Tables

**Table 1:** Comparison of clinical characteristics in the 3D-MODEL and EAM groups.

	3D-MODEL Group (*n* = 149)	EAM Group (*n* = 79)	*p*-Value
Female	96 (64%)	47 (59%)	0.46
Age (years)	50 ± 17	49 ± 15	0.60
Weight (kg)	76 ± 14	76 ± 14	0.76
Height (cm)	167 ± 9	168 ± 9	0.58
BMI	27 ± 5	27 ± 5	0.76
LVEF (%)	61 ± 7	60 ± 8	0.32
LA diameter (mm)	36 ± 6	38 ± 6	0.59
Hypertension	29 (37%)	62 (42%)	0.47
Diabetes mellitus	12 (8%)	5 (6%)	0.63
Hyperlipidemia	54 (36%)	25 (32%)	0.49
B-blocker	84 (56%)	50 (63%)	0.31
Propafenone	18 (12%)	10 (13%)	0.9
Amiodarone	2 (1%)	3 (4%)	0.23
Sotalol	16 (11%)	12 (15%)	0.33

**Table 2:** Comparison of procedural characteristics in 3D-MODEL and EAM groups.

	3D-MODEL Group (*n* = 149)	EAM Group (*n* = 79)	*p*-Value
Procedural time (min)	38 ± 14	80 ± 39	<0.0001
Number of applications	5 ± 4	9 ± 8	<0.0001
RF time (s)	251 ± 176	503 ± 425	<0.0001
Max power (W)	41 ± 8	36 ± 8	<0.0001
Max temperature (°C)	61 ± 13	41 ± 9	<0.0001
Fluoroscopy time (s)	284 ± 167	260 ± 327	0.49
PVC/VT from septal RVOT	123 (83%)	48 (61%)	0.001
PVC/VT from RVOT free wall	26 (17%)	28 (37%)	0.001
Open-irrigated ablation catheter	14 (9%)	62 (78%)	0.0001
PVCs	138 (93%)	67 (85%)	0.06
VT/VT	11 (7%)	12 (15%)	0.06
Acute success in PVC group	123 (89%)	54 (81%)	0.10
Acute success in VT group	10 (91%)	11 (92%)	0.95

## Data Availability

The data presented in this study are available upon request from the corresponding author.
